# Recognition of Billy Day for service to reproductive biologists

**DOI:** 10.1093/biolre/ioad056

**Published:** 2023-06-12

**Authors:** James E Kinder

**Affiliations:** The Ohio State University, Columbus, OH 43210; CQUniversiy, Rockhampton, Qld 4701, Australia


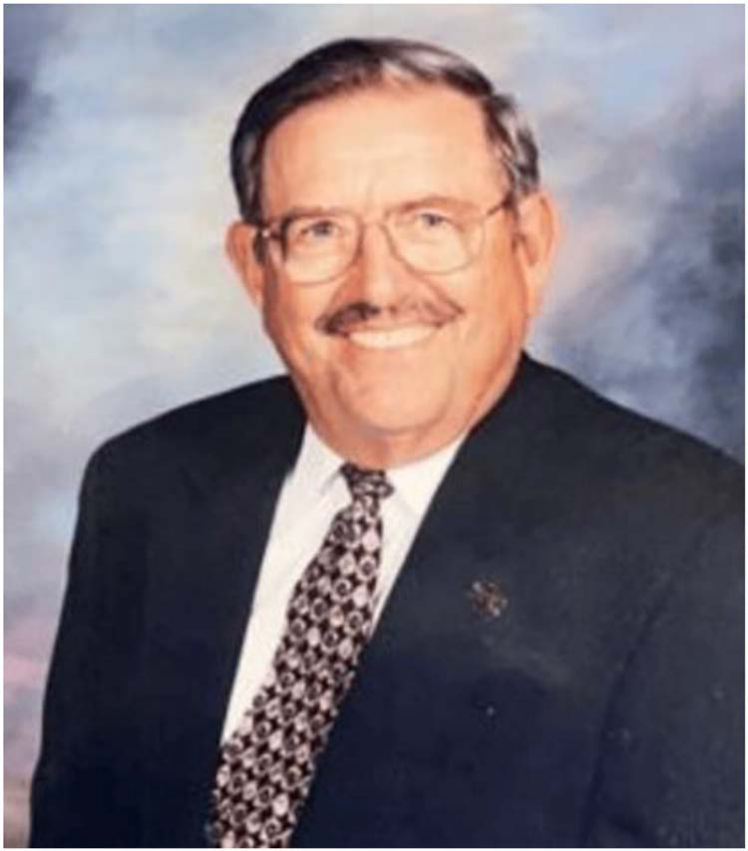


It was with great sadness when we learned of the recent passing of *Dr. Billy Day*, long time member of SSR. Because of my long-standing relationship with Billy that began when I was an undergraduate student at the University of Missouri and continued throughout the rest of my career, I was asked by Dr. Jennifer Wood, in her leadership role with SSR, to provide my reflections in a tribute to Billy. I also appreciate the input of Dr. Billy Flowers in drafting these reflections. Dr. Flowers worked with Billy on his graduate program and provided some important insights into Billy’s contributions in mentoring students. Family was extremely important to Billy. As a native West Virginian, Billy maintained strong ties with his family in the Petersburg area throughout his entire life. He earned his BS and MS degrees from West Virginia University (WVU) where he was formally recognized as a distinguished alumnus. While at WVU he met the love of his life, his wife Anabelle, who always championed his lifetime endeavors. Subsequently, Billy completed his PhD at Iowa State where he has been recognized also as a distinguished alumnus. At Iowa State, Dr. Bob Melampy, served as his mentor. I listened to Billy reflect on Dr. Melampy many times, and it was obvious he had a profound impact on Billy’s approach to research and in his engaging way of working with peer reproductive biologists.

Billy subsequently joined the faculty at the University of Missouri (Mizzou) in 1958 where he mentored many who went on to be leaders in the field of reproductive biology. Two of his greatest research contributions were his and his graduate student’s elucidation of the most efficacious management of media for in vitro fertilization and in utilization of prostaglandin F_2α_ for induction of parturition in pigs. I first met Billy at Mizzou as an undergraduate student. He was not only a great teacher of reproductive physiology, but also about “life in general” for his students. He was integral in my career choice to become a reproductive biologist and was instrumental to my entrance into graduate school. I was from a rural region in southeast Missouri and was not prepared to go to college. For some reason, I expect because of our similar backgrounds before college, Billy saw the scientific potential in me at this early stage of my academic career. We remained in close contact with each other during my graduate programs, and he served as a lifetime mentor for me and many of those who were his students at Mizzou.

I remember one of Billy’s favorite sayings was that “…you should always leave something better than you found it…” and it is evident that he took this to heart during his career at Mizzou. He was the visionary creator, builder, and sustainer of the internationally renowned reproductive biology program at the University of Missouri. He was instrumental in gaining state funding for the innovative Food for 21^st^ Century Program which provided the initial resources for the establishment of the present-day Mizzou reproductive biology program. I also remember how Billy had a clear vision of the type of facility in which research should be conducted and this vision subsequently became the Mizzou Animal Sciences Center which provided the physical infrastructure to support the scientists and students associated with this internationally renowned reproductive biology program. From my perspective, Billy truly was the cornerstone upon which the present-day elite Mizzou reproductive biology program was built. In conversations with Billy over the years, I came to understand his vision for the present-day program and facilities at Mizzou that was “conceived” in the mid-1960s when he was housed in the humble infrastructure of Mumford Hall - which was surrounded by “temporary” buildings constructed for housing the military personnel during World War II.

Billy valued the many recognitions for his great accomplishments in reproductive biology. The one for which he was the proudest, however, was the Fred F. McKenzie Distinguished Professor of Animal Reproduction. Billy always upheld Dr. McKenzie as an elite reproductive biology researcher, who preceded Billy as a faculty member at the University of Missouri, and who was recognized for his superb graduate student mentorship, one of which was Dr. L.E. Casida, who himself went on to have a highly distinguished career as a graduate student mentor at the University of Wisconsin. Other recognitions of Billy’s contributions to the field of reproductive biology and animal sciences are the most prestigious awards in the field - Physiology and Endocrinology and Morrison Awards, respectively, from the American Society of Animal Science the latter of which is the highest honor bestowed upon its members. Billy was very proud of his recognition with the Society of the Study of Reproduction award for lifetime contributions to reproductive physiology and always taught his students that “…reproduction occurs in all animals not just the one you happen to be working with at the moment…”.

As a mentor, I consider Billy as the “Master of the Teachable Moment” in the explicit and direct way he communicated his thoughts when I asked him for advice. I remember numerous settings where these communications occurred, one being in his office at the University Missouri on a weekend afternoon. I was expressing concern about how my major professor for my PhD program at Washington State, Jerry Reeves, had what I considered too great expectations of me in excelling as a researcher; being an excellent classroom student; in my graduate teaching endeavors; service to the department; and in assisting my peers with their projects. What came next, as many of his students probably remember, was the “bucket talk” as Dr. Flowers describes it in which Billy emphasized that we all have many different buckets that we have to carry at the same time, and some are heavier than others. What Billy was really telling me emphatically was, “Jerry is preparing you for the future and you need to figure out how to deal with it by setting priorities because doing so will be a mark of success in your career”. Billy emphasized that we all have 24 hours in a day and that it was up to me to determine my priorities in ways that I could be successful.

I also remember Billy’s advice when I was considering whether to take an 85% teaching position at Ohio State in the mid-1970s or do a postdoctoral with Andy Schally, soon to be recognized as a Nobel Laureate. I thought for sure Billy would encourage me to go work with Schally but he didn’t - rather he said go to Ohio State - “Do a good job and somebody else will want you”. Several years later, after having done a “good job” at Ohio State and having been employed in a position at the University of Nebraska, Lincoln (UNL), I was being encouraged to take on a greater teaching and lesser UNL research role. I attempted to contact Billy one evening via the phone for his advice and Anabelle answered and told me Billy was quite ill, and I responded I’ll get back to him later. I, however, heard Billy in the background as I was about to hang up (no cell phones at that time) and he asked Anabelle who it was and, even though quite ill, he made it a priority to visit with me. I told him of what was occurring at UNL regarding the request that had occurred. He immediately responded – “You are just beginning your research career and if you want to get the satisfaction from mentoring graduate students, you need to continue with a significant emphasis on research”. I guess he thought I did well in considering his advice because a year or so later he advised his son, Mike, to come and work with me on his graduate student endeavors at UNL. Mike chose to do so and is presently Department Chair at Kansas State University after a highly productive career in teaching and research at The Ohio State University.

My final reflection of Billy as a mentor that I include in these remembrances is a morning when I was having breakfast with him in Columbia – the home of “Ol Mizzou”. There was a man who stopped by the table and he and Billy had a short visit. When the man walked away, Billy looked at me and said he was head groundskeeper at the University of Missouri for many years and was the epitome of how those in a university should function - as “servants to others, particularly the students”. In all these and many other occurrences, Billy Day had a great impact on my decision making and manner of functioning in my career, similar to how he did with many others in his distinguished service as a faculty leader in the field of reproductive biology and successes of those with whom he worked and mentored. I frequently tell my students that determining who to listen to in life and who not to is a primary factor as to whether one is successful and gets satisfaction in their life’s endeavors. One of the wisest decisions I ever made was to make it a priority to engage with Billy Day as my mentor.

In visiting with Dr. Joy Pate, longtime SSR leader, last week about Billy’s passing, she made the comment - “I always remember how Billy Day greeted me with that infectious smile”. I reflected on her thought and realized how true it indeed was - a very effective way, as evident in the picture shared with this article, in which he invited Joy, me, and many others to have conversations that markedly affected our treks down life’s pathway.

